# Continuous Change in Membrane and Membrane-Skeleton Organization During Development From Proerythroblast to Senescent Red Blood Cell

**DOI:** 10.3389/fphys.2018.00286

**Published:** 2018-03-26

**Authors:** Giampaolo Minetti, Cesare Achilli, Cesare Perotti, Annarita Ciana

**Affiliations:** ^1^Laboratori di Biochimica, Dipartimento di Biologia e Biotecnologie, Università degli Studi di Pavia, Pavia, Italy; ^2^Servizio Immunoematologia e Medicina Trasfusionale, Fondazione IRCCS Policlinico San Matteo, Pavia, Italy

**Keywords:** artificial red blood cells, reticulocyte maturation, red blood cell ageing, multivesicular endosome, membrane rafts, fluid phase endocytosis, autophagy, spleen

## Abstract

Within the context of erythropoiesis and the possibility of producing artificial red blood cells (RBCs) *in vitro*, a most critical step is the final differentiation of enucleated erythroblasts, or reticulocytes, to a fully mature biconcave discocyte, the RBC. Reviewed here is the current knowledge about this fundamental maturational process. By combining literature data with our own experimental evidence we propose that the early phase in the maturation of reticulocytes to RBCs is driven by a membrane raft-based mechanism for the sorting of disposable membrane proteins, mostly the no longer needed transferrin receptor (TfR), to the multivesicular endosome (MVE) as cargo of intraluminal vesicles that are subsequently exocytosed as exosomes, consistently with the seminal and original observation of Johnstone and collaborators of more than 30 years ago (Pan BT, Johnstone RM. Cell. 1983;33:967-978). According to a strikingly selective sorting process, the TfR becomes cargo destined to exocytosis while other molecules, including the most abundant RBC transmembrane protein, band 3, are completely retained in the cell membrane. It is also proposed that while this process could be operating in the early maturational steps in the bone marrow, additional mechanism(s) must be at play for the final removal of the excess reticulocyte membrane that is observed to occur in the circulation. This processing will most likely require the intervention of the spleen, whose function is also necessary for the continuous remodeling of the RBC membrane all along this cell's circulatory life.

“In fact, it may almost be true to say that the red cell continuously changes until its senescent properties are recognized and it is eliminated from the circulation.”

(Ahn and Johnstone, 1991)

## Introduction

Recapitulation of erythroid differentiation and amplification *in vitro* is an actively investigated topic for the potentially revolutionary impact on regeneration and transfusion medicine, for all the conditions where reintegration of functional blood volume is required. Significant advancements in the field have made it possible to expand and differentiate *in vitro* erythroid precursors to the stage of enucleation, starting from progenitor cells of various origin: embryonic stem cells (Lu et al., 2008; Ma et al., 2008); adult circulating CD34^+^ cells (Migliaccio et al., 2002; Neildez-Nguyen et al., 2002; Giarratana et al., 2011; Griffiths et al., 2012b); CD34^−^ mononuclear cells (MNC) (van den Akker et al., 2010; Tirelli et al., 2011); umbilical cord blood (Neildez-Nguyen et al., 2002; Leberbauer et al., 2005; Miharada et al., 2006; Baek et al., 2008; Kupzig et al., 2017) or even immortalized cell lines (Hiroyama et al., 2008; Trakarnsanga et al., 2017).

Paramount for the possibility of developing a simplified *in vitro* culture of RBCs was the discovery that the last stage in erythroblast development, the expulsion of the nucleus, is an intrinsic property of the erythroblast that does not require an erythroblastic island to occur. In fact, the central macrophage in the island appears to be actively involved, at this stage, only in the phagocytosis of the extruded nucleus (Koury et al., 1988; Qiu et al., 1995; Ji et al., 2010). Although it is possible that significant scale-up will require co-culture with bone marrow stromal cells (Giarratana et al., 2005; Mountford et al., 2010; Rousseau et al., 2014), to date differentiation to the reticulocyte (retic) stage can be obtained in liquid cultures using only soluble factors and it is reasonable that in a relatively short time the target concentration of 5 × 10^7^ cells/ml could be reached (Rousseau et al., 2014). Yet, full development of the retic to a mature biconcave RBC under artificial conditions appears to still be a major challenge (Giarratana et al., 2005, 2011; Koury et al., 2005; Miharada et al., 2006). A number of reviews are available on the erythroblastic island and erythroid differentiation (Mountford et al., 2010; An and Mohandas, 2011; Anstee et al., 2012; Griffiths et al., 2012a; de Back et al., 2014; Satchwell et al., 2015a; Mankelow et al., 2016; Moras et al., 2017). The present article reviews the literature on retic maturation, one of the unsolved problems of erythropoiesis (Chasis et al., 1989; Koury et al., 1989; Blanc and Vidal, 2010; Ney, 2011), seen as a continuum of differential remodeling of the lipid bilayer and of the membrane-skeleton that leads to the mature circulating RBC first and then to the senescent RBC, and focusing on aspects of membrane topology and selectivity of protein sorting.

## Sorting of proteins during enucleation

In early seminal work, synthesis of RBC membrane proteins was described to occur asynchronously in avian and murine RBCs (Lazarides and Moon, 1984; Hanspal and Palek, 1987; Hanspal et al., 1992). In murine erythroblasts, band 3 synthesis, through a complex dynamics of trafficking from internal membranes to the plasma membrane of dimeric and tetrameric forms of the protein (Hanspal et al., 1998), was completed while the translation of spectrins was still in progress. More recent studies of human erythropoiesis have revealed that band 3 is synthesized early (Gautier et al., 2016) and assembled into multiprotein complexes, especially with protein 4.2, in the intracellular compartment (Satchwell et al., 2011). In a study (Chen et al., 2009) carried out with murine erythroblasts infected with the anemia-inducing strain of Friend erythroleukemia virus (FVA cells) (Koury et al., 1984), many RBC proteins were followed throughout maturation from proerythroblast to retic. Three patterns of protein turnover were observed for integral proteins. The levels of some proteins, including band 3 and glycophorin A (GPA), increased toward the late orthochromatic stage, those of others (CD44, Lu, ICAM-4, and β1 integrin) decreased throughout differentiation, and still others, among which the transferrin receptor (TfR or CD71), remained stable. Of the membrane-skeletal proteins all were shown to increase steadily except for actin, which decreased from proerythroblast to retic (Chen et al., 2009). Similar results were obtained with human erythroblasts (Hu et al., 2013). Comparative transcriptome and expression analysis of human and murine erythroid differentiation (An et al., 2014; Satchwell et al., 2015a; Gautier et al., 2016) is beginning to shed more light on this developmental program and its interspecies differences, which are responsible for generating the heterogeneous phenotypes seen in RBCs of different mammals (Ahn and Johnstone, 1991). Moreover, additional useful information would come from analysis of retic transcriptome, evaluated at different stages of retic maturation (Goh et al., 2007; Malleret et al., 2013).

**Figure 1:**
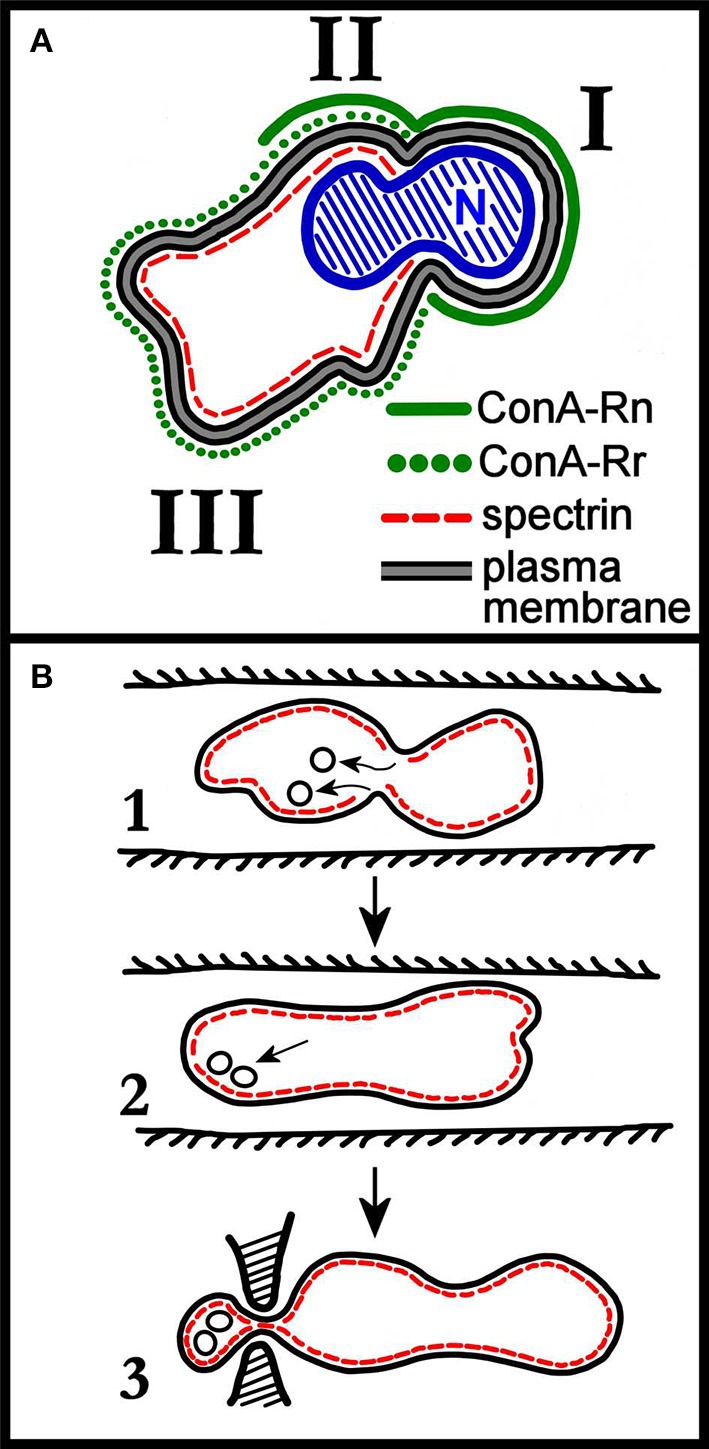
**(A)** Schematic distribution of membrane proteins in the enucleating mouse erythroblast. Roman numerals I, II, and III correspond to the domains identified by the authors of the work (Geiduschek and Singer, 1979). Domain III corresponds to the incipient reticulocyte. The nucleus (N), not completely extruded, shows the characteristic constriction which separates domain I from domain II. As discussed in the text of the cited work, two classes of ConA receptors (ConA is a lectin that binds to band3, so ConA receptors are band 3 molecules) are assumed to be present in the erythroblast membrane, with ConA-Rn (but not ConA-Rr) present in domain I; both ConA-Rn and ConA-Rr in the membrane of domain II; and ConA-Rr (but not ConA-Rn) in domain III. Most strikingly, spectrin is confined to domains II and III. Re-drawn from Geiduschek and Singer (1979). **(B)** Rudimentary early depiction of a model for the remodeling of the cell membrane during retic maturation to RBC *in vivo*. Panel 1 depicts the invaginations of spectrin-free domains of the retic membrane occurring in the circulation, and their subsequent endocytosis. In panel 2, the endocytosed vesicles are pictured as associating with the membrane either to fuse with it or to be exocytosed. In panel 3, the exocytosis of vesicles in a larger, spectrin-containing body is pictured as occurring by mediation of the spleen. Re-drawn from Zweig et al. (1981).

Whatever the set of membrane proteins with which developing erythroblasts arrive at the orthochromatic stage, this composition will undergo a major redistribution with the enucleation event. This concept was already clear in early seminal studies carried out mostly in mice. Concerning transmembrane proteins, in enucleating mouse erythroblasts isolated from bone marrow, band 3 was shown to partition at a higher concentration in the membrane surrounding the nucleus, than in the membrane of the incipient retic. On the other hand, GPA, the second most abundant integral protein of the RBC membrane, was found to be equally distributed between these two domains of the membrane at enucleation (Figure 1A; Geiduschek and Singer, 1979). In erythroblasts isolated from rat bone marrow a similar partition pattern was described (Skutelsky and Farquhar, 1976).

More recently, GPA was found to partition almost exclusively to the retic during enucleation of FVA cells, possibly because of transiently increased connectivity, of unknown origin, of GPA to the spectrin skeleton (Lee et al., 2004). It should be observed that the cell model adopted may influence the results obtained. Many studies were carried out with FVA cells that may not exactly represent physiological erythroid differentiation. Nevertheless, this scenario was largely confirmed in a recent proteomic study of protein distribution during human erythroblast enucleation, although e.g., the restriction of band 3 to the retic membrane seemed to be less defined than what seen in the murine model (Bell et al., 2013).

Concerning membrane-skeletal proteins, they all partition to the retic by a remarkably selective sorting process whose mechanism remains unknown (Geiduschek and Singer, 1979; Zweig et al., 1981; Wickrema et al., 1994; Bell et al., 2013).

## Sorting of proteins during Retic-to-RBC maturation

A major remodeling of the reticulocyte in the maturation to RBC entails the loss of approximately 20% plasma membrane and the continued removal of residual intracellular components. Concerning membrane-skeletal proteins, they are thought to be largely retained in the maturing RBC through retic-to-RBC transition (Tokuyasu et al., 1979; Pan and Johnstone, 1983). A recent study on stress murine retics documents this in detail (Liu J. et al., 2010). Here all the main membrane-skeletal proteins (α-spectrin, β-spectrin, ankyrin R, protein 4.1R, membrane-associated actin, protein 4.2, p55, tropomodulin, protein 4.9) were found to be unchanged between retics and RBCs. Other cytoskeletal components declined (myosin, tropomyosin, adducin) and some of them were completely lost (tubulin, cytosolic actin). Concerning transmembrane proteins, a decline of Na^+^/K^+^ATPase, NHE1, GPA, CD47, Duffy, and Kell was observed, together with the complete loss of CD71 and of ICAM4. Interestingly and surprisingly, the following proteins were found to be increased in the membrane of the mature RBC: band 3, GPC, Rh, RhAG, XK, and GPA (for GPA, only the subset of molecules that act as receptor for the TER-119 antibody; Kina et al., 2000). This phenomenon was not commented further in the cited paper (Liu J. et al., 2010) (for a plausible explanation see below). A more recent proteomic analysis of human retics obtained by *in vitro* expansion and differentiation in *in vitro* culture of cord blood-derived CD34^+^ cells, largely confirmed a similar pattern of protein partitioning between cultured retics and the autologous mature RBCs recovered from cord blood (Wilson et al., 2016).

## Models of reticulocyte maturation

The loss of retic's excess membrane was originally proposed to occur through the mechanism depicted in Figure 1B, for which hints were already available since the 1960's (Kent et al., 1966; Holroyde and Gardner, 1970). Here the excess surface area first invaginates as endocytic spectrin-free vesicles, which are then hypothetically removed as the content of a larger spectrin-containing vesicle, possibly with the active intervention of the spleen. Later, Johnstone et al. (Pan and Johnstone, 1983; Johnstone, 2005) and Stahl and co-workers (Harding et al., 1984, 2013) almost at the same time discovered that in maturing retics a special endosomal compartment is formed, named multivesicular endosome or body (MVE, MVB), which contains intraluminal vesicles (ILVs) carrying the TfR and other selected transmembrane proteins, but lacking band 3 and GPA. Fusion of the MVE with the plasma membrane and exocytosis of the ILVs is the process though which maturing retics dispose of no longer needed membrane proteins. Although the discovery by Johnstone and co-workers was seminal for subsequent understanding of endocytic processes and vesicular trafficking and may explain the loss of most of the TfR, it does not account entirely for the loss of membrane surface area from retic to mature RBC (see below).

According to the current view of the vesicular trafficking around the complex endosomal compartment, several endocytic processes exist in eukaryotic cells, clathrin-dependent and -independent, for the purpose of captating extracellular components and for the internal needs of the cell of turning over plasma membrane constituents. Thus, the endocytic vesicles produced by clathrin-mediated endocytosis, caveolar endocytosis, flotillin-dependent endocytosis and Arf6-dependent endocytosis all fuse generating a common compartment, the early (sorting) endosome (Otto and Nichols, 2011; Bohdanowicz and Grinstein, 2013; Johannes et al., 2015). From the sorting endosome, a MVE can form that can take two different routes: one is fusion with the lysosome for complete hydrolytic degradation of the ILVs; the other is fusion with the plasma membrane for discharging the ILVs, that can now be defined as exosomes (Cocucci and Meldolesi, 2015), in the extracellular space.

Topologically, the orientation of transmembrane proteins in ILVs and exosomes is the same as in the plasma membrane (right-side-out) and as in ectosomes (intended as vesicles released extracellularly directly from the plasma membrane), whereas it is opposite in endocytic vesicles (inside-out).

There are therefore two steps at which proteins must be sorted along the above-described pathway. The first entails the selection at the plasma membrane for endocytosis. In the specific of retic maturation, endocytosis of TfR from the plasma membrane. The next sorting step occurs on the membrane of the sorting endosome where the TfR must be again selected among other membrane proteins to be inserted in ILVs. This process is mediated by a special molecular machinery based on ESCRT protein complexes (Gruenberg and Stenmark, 2004; Kowal et al., 2014) although an ESCRT-independent, ceramide-based mechanism has also been described (Trajkovic et al., 2008).

Concerning the sorting of plasma membrane proteins, as mentioned in the previous section, an increase in band 3 and GPC was observed in the maturation of mouse retics to RBCs by Western blotting of whole cell proteins, normalizing the quantity of sample by loading the same amount of cholesterol for RBCs and retics (Liu J. et al., 2010). Whereas, a decline of proteins during the retic-to-RBC transition can be easily accepted, their stability or increase in a membrane that is at the same time decreasing in size is less easily understood, especially for a complex multi-pass transmembrane protein like band 3. This is certainly not due to *de novo* synthesis in a cell that is progressively dismantling its internal translational apparatus and consuming the molecular machinery required for vesicular trafficking and where band 3 was synthesizes and already partially assembled in complexes in earlier erythroblastic stages (van den Akker et al., 2011). As the decrease in membrane surface area that occurs in the retic-to-RBC maturation is of the same order of magnitude as the observed “increase” in band 3 and GPC, the latter can only be explained by the increase in concentration that follows a selective loss of cholesterol (and other components different from band 3) by the retic in the process. Previous and independently obtained evidence exists on stable amounts of band 3 in retics and RBCs, despite the large difference in surface area between the two cell types (Foxwell and Tanner, 1981), pointing to a peculiar and as yet unknown mechanism for such selective retention of band 3 in the membrane of maturing retics that must be capable of discriminating between two almost equally abundant transmembrane proteins, band 3 with approximately 1.2 × 10^6^ copies/cell and CD71 with approximately 8 × 10^5^ copies/cell (Van Bockxmeer and Morgan, 1979) and completely get rid of the latter while largely saving the former.

To explain the selective retention in the reticulocyte membrane of such abundant transmembrane proteins as band 3, GPA, RhAG, GPC, CD47, a mechanism based on the formation of stable multiprotein complexes among the above-mentioned species, with the association to components of the membrane-skeleton (Satchwell et al., 2015b, 2016) could be conceived. This mechanism would work by mechanically preventing incorporation of the bulky protein complexes into endovesicles. However, this is difficult to reconcile with the evidence that, for instance, some subpopulations of band 3 molecules or even band 3 complexes (with 4.2 and ankyrin), may still move around unanchored to the skeleton in retics (due to the extra membrane present at this stage, that cannot be supported by a membrane-skeleton of smaller area). Moreover, even in fully mature normal RBCs there is a significant 30% of band 3 molecules which is highly mobile, being not anchored to the skeleton. This population of band 3 molecules should be in principle free to leave the cell if size reduction was driven by a non-specific mechanism, but it is not: band 3 (as well as GPA and GPC) are not found in exosomes derived from ILVs in MVEs, as discovered already by Johnstone and co-workers.

There is actually one particular experimental model in which a peculiarly sharp segregation of different membrane proteins is observed, and that is during the isolation of membrane rafts as detergent resistant membrane (DRM). When DRMs are properly isolated from RBCs, they meet the canonical requirements for membrane rafts of being enriched in cholesterol, sphingolipids and specific classes of proteins like GPI-linked proteins, flotillins and stomatin, to name only the most relevant. In addition, and strikingly, they are completely devoid of band 3 and GPC (Ciana et al., 2005, 2011, 2013, 2014; Crepaldi Domingues et al., 2009; Domingues et al., 2010; Achilli et al., 2011; Minetti et al., 2013).

We infer from literature data and from our own experimental evidence that the step at which band 3, GPC, and GPA (Johnstone, 1992) are selected for being retained in the plasma membrane at the retic-to-RBC transition is in the trafficking of MVEs, and implies a mechanism based on differential partitioning of a given transmembrane protein into the membrane raft phase. To the best of our knowledge this is the first time that a membrane raft-based process for the sorting of membrane proteins during retic maturation is proposed.

**Figure 2:**
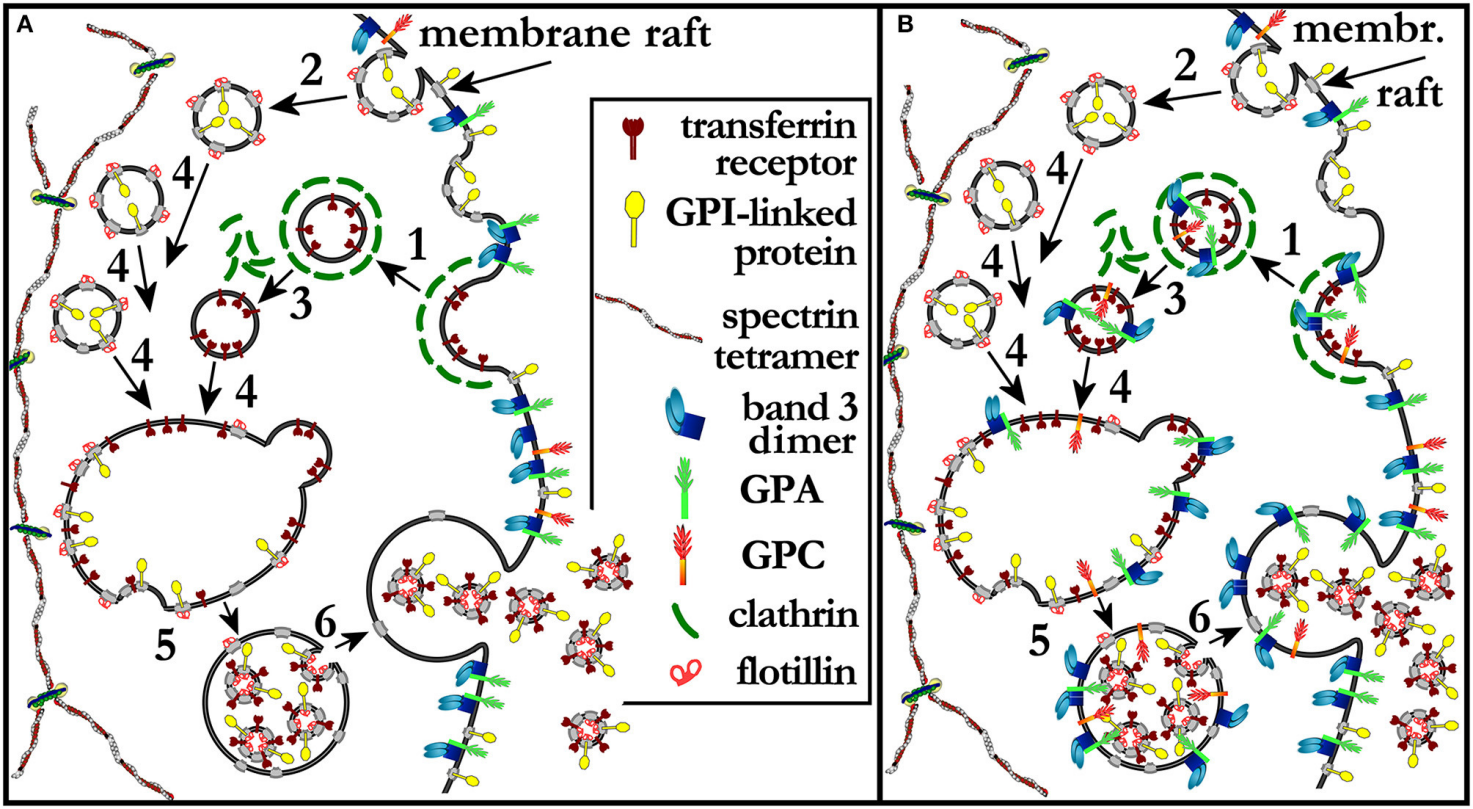
**(A)** A model extended from that proposed by Johnstone et al. (Johnstone, 2005). Reticulocytes mature through the release of TfR-containing exosomes from MVEs. Clathrin coated vesicles and lipid rafts have been added to the original scenario, to show that TfR is recruited at clathrin-coated pits and endocytosed in clathrin-coated vesicles (1) that do not contain membrane rafts nor band 3, GPC, or GPA. Parallel routes of endocytosis must exist that deliver membrane raft components to the MVB while, again, excluding band 3, GPC and GPA (2). After shedding of the clathrin coat (3) from clathrin-coated vesicles, all vesicles converge and fuse (4) in a single early endosome, which is soon converted into a MVB with the endovesiculation of ILVs that contain both membrane raft components and TfR (5). The membrane of the MVB becomes thus depleted of TfR and membrane raft constituents, including cholesterol and sphingolipids, The MVB eventually fuses with the plasma membrane and releases the ILVs that are now defined as exosomes. The bulky structure of the spectrin skeleton is depicted as separated from the scenario where this vesicular trafficking occurs. In **(B)** a different pathway is proposed whereby band 3, GPC, and GPA can reach the MVB membrane coming from, for instance, clathrin-coated vesicles, but then are not packaged into ILVs and exosomes because of their inability to partition to the raft phase. Band 3, GPC, and GPA are therefore returned to the plasma membrane with the fusion of the MVB with it. See text for additional details.

At the plasma membrane the TfR is selected as the cargo of clathrin-coated vesicles with the mediation of the adaptin AP-2 (Liu A. P. et al., 2010; Kelly and Owen, 2011). The same route of entry is contemplated in early work of Johnstone (1992). It is accepted notion that clathrin-mediated endocytosis occurs at regions of the membrane that exclude lipid rafts, whereas caveolin-mediated and possibly other forms of endocytosis include micro- nano-domains of typical raft composition (Rajendran and Simons, 2005). In a recent study on mesenchymal stem cells, lipid rafts and TfR were found to co-localize in the MVB membrane, and it was concluded that they were ferried there together in the same clathrin-coated vesicles that were endocytosed from the plasma membrane (Tan et al., 2013). However, because of the accepted notion that rafts do not partition in clathrin-coated vesicles, it is more likely that diverse components of the plasma membrane converge in a single MVB coming from different endocytic routes. Plenty of evidence indeed exists on the presence of membrane raft markers in the exosomes. GPI linked proteins (a typical raft marker) were detected in TfR-containing exosomes already by Johnstone (1992); in exosomes released *in vitro* by rat “stress” retics, and also from retics from human patients, canonical raft markers such as flotillin, stomatin, ganglioside GM1 were found enriched (de Gassart et al., 2003). All this clearly suggests that a pathway must exist, different from the clathrin-coated mediated one, for delivering raft material to the MVB first and then to the exosomes (the scenario described above is depicted in Figure 2A). Incidentally, these other raft-dependent endocytic vesicles could be responsible for the disposal of other transmembrane proteins that do not follow the clathrin-dependent pathway. Unfortunately, in the cited studies the membrane of the MVB was never characterized for the presence of band 3, GPA or GPC, so the possibility has never been evaluated that band 3 (plus GPC and GPA) actually reaches the MVB via any of the various endocytic pathways, but then it is excluded from the ILVs because the discriminating step occurs at the level of the budding of ILVs inside the MVB. At this latter step, the selection mechanism could be based on different affinities of a given protein for the raft phase (maybe exploiting the ESCRT-independent route mentioned above; Trajkovic et al., 2008). According to this mechanism, band 3 would not be inserted in exosomes, but will be entirely recycled to the plasma membrane at the time the MVB fuses with it (this alternative scenario is depicted in Figure 2B). Experimentally answering this question would be important to understand the selectivity of the whole process. It would also clarify whether the exclusion of band 3, GPC, and GPA from membrane rafts is due to an intrinsic property of these proteins, and what is the role played by the membrane-skeleton in this scenario.

Along this line, a number of other issues still have to be solved concerning the maturation of retics to RBCs. What is the role played by the membrane-skeleton when, after enucleation, in the immature retic the heavy intracellular vesicular traffic must efficiently endocytose and circulate endocytic vesicles to generate the MVEs and then move the latter to the plasma membrane for the final fusion and the release of exosomes? Is this taking place because the membrane-skeleton is still completely or partially unanchored to the bilayer? Or is the spectrin network temporarily detached from the bilayer only locally where endocytic vesicles form? The first scenario is more likely, because portions of the retic membrane must be still floating unattached to the membrane-skeleton, making it more likely to be a site where endocytic formation is not hindered by a bulky spectrin network. In this respect, there appear to be significant differences in the ontogeny of primitive and definitive erythroblasts, whereby, at least in the mice model, primitive erythroblasts enucleate intravascularly, after having circulated for some time without a fully assembled membrane-skeleton (therefore with a less deformable cell structure). In definitive erythropoiesis, instead, erythroblasts do not enucleate until just before leaving the bone marrow and circulate with a stable membrane-skeleton, and therefore with higher deformability, although with an excess lipid bilayer that must be shed by the circulating retic. In both cases, for stable membrane-skeleton assembly, a switch to the expression of the mature spliceoform of protein 4.1R appears to be fundamental (Huang et al., 2017). However, the timing and modalities for the establishment of a stable membrane-skeleton in erythropoiesis before and after enucleation are still poorly understood.

Another possible trafficking pathway has been recently described adding complexity to the process of retic maturation. Here, endovesiculation (not better characterized as being or not clathrin-dependent) would produce GPA-positive endosomes that would then fuse with a late autophagosome. The latter, in turn, will be “extruded” from the cell by “crossing” the plasma membrane while maintaining its topology. Through this process it would be possible for the retic to eliminate additional membrane area as large vesicles, which are phosphatidylserine-positive because of their inside-out nature. Curiously, the fate of TfR was not followed in these works, so it is not possible to ascertain whether it is also part of the membrane that is lost by this mechanism. Several topological aspects, however, remain poorly defined and render this membrane trafficking route difficult to comprehend (Griffiths et al., 2012a,b; Mankelow et al., 2016).

It is interesting to note that the TfR was very likely recycled, together with transferrin, through several rounds of receptor-mediated-endocytosis for iron captation during differentiation from proerythroblast, whereas, at the retic-to-RBC transition it is instead routed to the MVE for eventual discharge from the cell. It is still a mystery how the cell can perform this switch in the routing of the same type of cargo, the TfR, at different stages of cell differentiation. It is also still unclear what is the relative contribution of the exosome pathway and other, possibly spleen-mediated pathways for the removal of excess TfR and membrane extension at the various stages of retic maturation.

Concluding on the relative enrichment in band 3 in the membrane of the mature RBC with respect to that of the retic (Liu J. et al., 2010), this would result from the selective loss of cholesterol as part of the membrane raft phase that is selectively eliminated with the exosomes. As seen in Figure 2, in fact, a profound imbalance in the composition of the lipid bilayer that is returned to the plasma membrane (when the MVB fuses with it) with respect to the membrane that is lost as exosomes, is generated along this membrane trafficking route. This hypothesis predicts, and to the best of our knowledge there are no data in the literature that answer this question, that the lipid composition of the plasma membrane should also change during maturation from retic to RBC, with a decrease in the ratio of (sphingolipids + cholesterol) to glycerophospholipids, and a decrease in membrane-raft associated proteins.

Importantly, the enrichment in band 3 described above as based on raft trafficking and previous evidence obtained on mature RBCs concerning the complete absence of band 3 and GPC from the DRM fraction, corroborates the long debated and never settled hypothesis (Sonnino and Prinetti, 2013) that DRMs are a good representation of membrane rafts (Ciana et al., 2014).

## Conclusions

It is unlikely that apparently autonomous mechanisms like the MVE-based trafficking or others like the ones involving autophagy, are the only processes through which retics eliminate their excess membrane. The reason for this is 2-fold: first, it is unclear for how long these mechanisms could be sustained in a cell that is progressively losing its molecular components, including the ones that organize MVE or autophagosome assembly (Gruenberg and Stenmark, 2004). In fact, at the same time that the plasma membrane is remodeled, additional processes are at play for the disposal of intracellular soluble components, likely through proteolytic processes including the ubiquitin/proteasome-mediated pathway, and residual organelles through autophagy and other routes (Moras et al., 2017). Ubiquitin was originally discovered in stress-retics from rabbit (Ciehanover et al., 1978), the ubiquitination/proteasome pathway is present and active to a very late stage (Liu J. et al., 2010; Neelam et al., 2011; Gautier et al., 2016), caspases and calpain are expressed in RBCs. The MVE-based mechanism was first described to occur in sheep stress retics, and it is probable that under physiological conditions it occurs predominantly in the bone marrow phase of retic maturation (R1). Maybe this could only be observed in circulating retics (R2) if they were the result of accelerated erythropoiesis and precocious egression from the bone marrow as it occurs in response to extensive phlebotomy or phenylhydrazine treatment. In fact, most of the TfR appears to be lost in R1 retics through the MVE pathway (Malleret et al., 2013), yet some is lost by circulating retics. Second, circulating retics require a functioning spleen for full maturation. It is therefore highly probable that under physiological conditions the MVE-based mechanism operates when retics are still in the bone marrow, and only partially, if at all, in R2 retics. Therefore, other as yet unidentified mechanisms should take over the duty of completing retic maturation. It is likely that this would imply the intervention of the spleen because the spleen is essential for the final steps in the topogenesis of a mature RBC membrane, with a biconcave discocyte shape and an optimized ratio of lipid bilayer to underlying membrane-skeleton. The spleen is also needed for the continuous processing of mature RBCs until they are cleared from the circulation (Crosby and Benjamin, 1957; Crosby, 1959; Kent et al., 1966; Holroyde and Gardner, 1970; Shattil and Cooper, 1972; Lux and John, 1977; Groom et al., 1991; Gifford et al., 2003).

The mechanism will also have to be capable of acting with some selectivity because residual TfR molecules will have to be removed from the surface of R2 retics, probably with a pinching action similar to that depicted in Figure 1B. Whether this is the actual mechanism and whether it also involves the recognition of membrane micro- nano-domains (membrane rafts) remains to be established.

On a methodological note, in times when the study of circulating micro-particles has become of age (Herring et al., 2013), assessing the origin of such entities is of importance for isolation and characterization. As RBC-derived micro-particles are usually recognized and isolated because they express GPA on their surface, any vesicles released by retics as exosomes would escape detection because exosomes originating from MVE appear not to contain GPA (Johnstone, 1992).

We have recently supported with novel experimental evidence the old notion that maturation and aging of circulating RBCs also require an intervention of the spleen (Ciana et al., 2017a,b; Kaestner and Minetti, 2017). In short, we have observed a disproportionate loss of flotillin-2 with respect to the loss in surface area that occurs during the ageing *in vivo* of normal human RBCs (approximately −17%). If this loss occurred through membrane vesiculation according to known spontaneous processes of ectosome release from RBCs, vesicles obtained *in vitro* from RBCs should contain flotillin-2 enriched with respect to the parent cell plasma membrane. Instead, we have observed the opposite. Moreover, we have observed that spectrin and other membrane-skeletal proteins decline with RBC ageing on a per-cell basis. This should not happen if ageing RBCs lost membrane as spectrin-free vesicles.

It should be noted that such splenic function may not be limited to a mechanical action on the RBC, but may require active recognition and removal of selected portions of the cell. To date, protocols for producing artificial RBCs succeed in the generation of significant amounts of enucleated erythroblasts. Complete maturation to a biconcave disc, however, has not been described to date to occur *in vitro* (Giarratana et al., 2011). It would be interesting to verify whether and to what extent the MVE-based mechanism is at play in artificially produced retics.

An advantage for transfusion medicine would be that, with the infusion of a cohort of synchronized “young” RBCs, the problem of the dramatic loss of up to 25% of stored RBCs in the first 24 h from transfusion should be solved or strongly attenuated. However, the infusion of blood units composed only of retics could pose an excessive burden on the recipient's spleen, which will have to process circulating retics at a much higher rate than the 1% retics per day that occurs under normal conditions. Therefore, full retic-to-RBC conversion may be required for clinical approval of artificial RBCs for transfusion purposes. It remains to be evaluated whether final maturation could be achieved by mimicking a mechanical splenic function or whether a fully biomimetic “artificial spleen” is required for this important maturational step.

## Author contributions

GM conceived the review and wrote a first draft, CA contributed to discussing the draft, to drawing the figures and writing the revised manuscript, CP discussed and contributed to writing the revised versions, AC conceived the manuscript and contributed to all revision phases with dicussions and writing of the text and figures.

### Conflict of interest statement

The authors declare that the research was conducted in the absence of any commercial or financial relationships that could be construed as a potential conflict of interest.
